# Optimizing pharmacotherapy and deprescribing strategies in older adults living with multimorbidity and polypharmacy: EuGMS SIG on pharmacology position paper

**DOI:** 10.1007/s41999-023-00872-0

**Published:** 2023-10-09

**Authors:** Eveline van Poelgeest, Lotta Seppala, Gülistan Bahat, Birkan Ilhan, Alpana Mair, Rob van Marum, Graziano Onder, Jesper Ryg, Marília Andreia Fernandes, Antonio Cherubini, Michael Denkinger, Annette Eidam, Angelique Egberts, Aðalsteinn Gudmundsson, Fatma Özge Kayhan Koçak, George Soulis, Jos Tournoy, Tahir Masud, Martin Wehling, Nathalie van der Velde, the EuGMS SIG Pharmacology

**Affiliations:** 1 Section of Geriatric Medicine, Department of Internal Medicine, Amsterdam UMC, University of Amsterdam, Amsterdam, The Netherlands; 2 Aging and Later Life, Amsterdam Public Health Research Institute, Amsterdam, The Netherlands; 3 Division of Geriatrics, Department of Internal Medicine, Istanbul Medical School, Istanbul University, Capa, Istanbul, Turkey; 4 Division of Geriatrics, Department of Internal Medicine, Liv Hospital Vadistanbul, Istanbul, Turkey; 5 Effective Prescribing and Therapeutics, Health and Social Care Directorate, Scottish Government, Edinburgh, Scotland, UK; 6 Edinburgh Napier University, Edinburgh, UK; 7 Aging and Later Life, Amsterdam Public Health Research Institute, Amsterdam, The Netherlands; 8 Department of Elderly Care Medicine, Amsterdam University Medical Centers, Location Vrije Universiteit Amsterdam, De Boelelaan 1117, Amsterdam, The Netherlands; 9 Fondazione Policlinico Gemelli IRCCS, Rome, Italy; 10 Università Cattolica del Sacro Cuore, Rome, Italy; 11 Department of Geriatric Medicine, Odense University Hospital, Odense, Denmark; 12 Geriatric Research Unit, Department of Clinical Research, University of Southern Denmark, Odense, Denmark; 13 Department of Internal Medicine, Hospital Curry Cabral, Centro Hospitalar Universitário de Lisboa Central, Lisbon, Portugal; 14 Geriatria Accettazione geriatrica e Centro di Ricerca per l’invecchiamento IRCCS INRCA, Ancona, Italy; 15 Agaplesion Bethesda Clinic Ulm, Institute for Geriatric Research, Ulm University, Geriatric Center Ulm, Ulm, Germany; 16 Center for Geriatric Medicine, Heidelberg University Hospital, AGAPLESION Bethanien Hospital Heidelberg, Heidelberg, Germany; 17 Department of Hospital Pharmacy, Franciscus Gasthuis & Vlietland, Rotterdam, Schiedam, The Netherlands; 18 Faculty of Medicine, Landspitali University Hospital, University of Iceland, Reykjavik, Iceland; 19 Division of Geriatrics, Department of Internal Medicine, Faculty of Medicine, Ege University, Izmir, Turkey; 20 Outpatient Geriatric Assessment Unit, Henry Dunant Hospital Center, Athens, Greece; 21 Hellenic Open University, Patras, Greece; 22 Department of Geriatric Medicine, KU Leuven University Hospitals Leuven Gasthuisberg Campus, Leuven, Belgium; 23 Department of Public Health and Primary Care, KU Leuven Biomedical Sciences Group, Leuven, Belgium; 24 Nottingham University Hospitals NHS Trust, Nottingham, UK; 25 Clinical Pharmacology Mannheim, Medical Faculty Mannheim, Heidelberg University, Mannheim, Germany

**Keywords:** Deprescribing, Polypharmacy, Older adults, Multimorbidity, Appropriate prescribing

## Abstract

Inappropriate polypharmacy is highly prevalent among older adults and presents a significant healthcare concern. Conducting medication reviews and implementing deprescribing strategies in multimorbid older adults with polypharmacy are an inherently complex and challenging task. Recognizing this, the Special Interest Group on Pharmacology of the European Geriatric Medicine Society has compiled evidence on medication review and deprescribing in older adults and has formulated recommendations to enhance appropriate prescribing practices. The current evidence supports the need for a comprehensive and widespread transformation in education, guidelines, research, advocacy, and policy to improve the management of polypharmacy in older individuals. Furthermore, incorporating deprescribing as a routine aspect of care for the ageing population is crucial. We emphasize the importance of involving geriatricians and experts in geriatric pharmacology in driving, and actively participating in this transformative process. By doing so, we can work towards achieving optimal medication use and enhancing the well-being of older adults in the generations to come.

## Key summary points

**Aim:**

To summarize the literature on medication review and deprescribing in older adults, and formulate recommendations to improve prescribing medications in older, multimorbid adults with polypharmacy.

**Findings:**

Current evidence demonstrates a need for a multifaceted and wide-scale change in education, guidelines, research, advocacy, and policy to improve the management of polypharmacy in older people, and to make deprescribing part of routine care for the ageing generations to come.

**Message:**

By implementing the recommendations in this paper, healthcare professionals will be better prepared to address the challenges associated with an ageing population and provide high-quality care to older patients with complex health and social care needs.

## Introduction

An ageing population means that more people will be living longer, but with multimorbidity [1, 2]. As a consequence, polypharmacy is becoming more prevalent, with two-thirds of adults over 65 years requiring multiple medications to manage their chronic conditions [3, 4]. While appropriate polypharmacy can be beneficial in managing symptoms and prolonging life, there is an increasing prevalence of inappropriate polypharmacy [5, 6]. This occurs when medicines are prescribed without evidence-based indication, are ineffective, or pose a risk for adverse drug reactions (ADRs). Potentially inappropriate medication (PIM) use is highly prevalent, not only in institutional care, but also in community-dwelling older adults [5–9].

There is a growing awareness of the potential harm associated with polypharmacy, especially in older adults [6, 10]. In fact, medication-related harm has recently been identified as a common geriatric syndrome [11]. Negative outcomes can include poor quality of life, drug interactions (drug–drug and drug–disease), poor medication adherence, increased morbidity, hospitalizations, increased length of hospital stay and mortality (drug related and overall), and greater economic burden [1, 6, 12–14]. In Europe, around 8.6 million unplanned hospital admissions are caused by ADRs annually, of which 70% are in older patients with polypharmacy [15]. To address this issue, the World Health Organization (WHO) established 'Medication Without Harm' as the third international Global Patient Safety Challenge in 2017 [16]. The initiative aims to raise global awareness about inappropriate prescribing and reduce serious avoidable medication harm by 50% globally within 5 years. In addition, medication safety is an important component of the global “Age-Friendly Health Systems” movement [17], and American recommendations for geriatric care [one of the four Ms (“matters most”) for stands for “Medication”]. There have been many national and regional initiatives aiming to improve safety in patients with polypharmacy, such as the Scottish Polypharmacy Guidance [18], the British CHARMER (CompreHensive geriAtRician-led MEdication Review) [19], and the recently launched SafePolyMed project (www.safepolymed.eu/).

For safe and effective pharmacotherapy in older adults, the medication list should be assessed for both over- and undertreatment and adjusted within the context of an individual patient’s care goals, current level of functioning, life expectancy, values, and preferences [3, 18]. Underprescribing occurs when effective treatments are not initiated despite a valid indication [7, 20], while overprescribing occurs when medications are prescribed without a valid indication or in inappropriate dosage, or interact unfavorably with certain patient conditions or concomitant medications. In practice, both over- and underprescribing are common [7, 20–22]. An important part of optimizing pharmacotherapy is deprescribing [23], the process of withdrawal of an inappropriate medication, supervised by a health care professional with the goal of managing polypharmacy and improving outcomes [24, 25]. It is a means to address and mitigate the current or potential harmful effects of inappropriate polypharmacy [24, 25], minimize drug-related harm for older adults, and improve their quality of life [3, 25]. Deprescribing can also reduce geriatric syndromes (such as cognitive decline [26] and falls [27]), pill burden [27–29], and the risk of hospitalization and death [27, 30]. On the other hand, deprescribing can also be harmful, as is suggested by studies in which antihypertensives were deprescribed in older patients, showing relevant—but not statistically significant—safety signals with more serious drug events (SAEs) in the intervention groups [31, 32]. This illustrates that deprescribing decisions should be weighed carefully. The recently published special issue of this journal by the European Geriatric Medicine Society (EuGMS) Task and Finish group on Fall Risk Increasing Drugs (FRIDs), “Deprescribing dilemmas in patients at risk of falls” (Volume 14, issue 4, August 2023), contains reviews on rational (de)prescribing of FRIDs.

This position paper, written on behalf of the (EuGMS Special Interest Group (SIG) on Pharmacology, discusses recommendations for the deprescribing component of optimizing pharmacotherapy in older adults with multimorbidity, and the role of healthcare professionals herein, with special focus on the role of geriatricians, and how to manage patient-centered polypharmacy in clinical practice. The paper also provides recommendations for future research efforts and implementation strategies, and reflects on deprescribing policy initiatives. Our recommendations are based on a (non-systematic) review of existing literature and expert knowledge. The expert group consisted of geriatricians, clinical pharmacologists, pharmacists, researchers, and policy makers from eleven countries. The co-authors reached consensus on the recommendations.

## Deprescribing in clinical practice

### The process of deprescribing

Medication review and deprescribing in older people with multimorbidity are challenging and not performed as frequently as it should [6]. Clinical inertia, possibly due to the difficulty of thoroughly evaluating the benefits and risks of medications in this population, may explain this [33]. While individual medications may be suitable based on guidelines for specific diseases, they may not be appropriate when considering multiple conditions, frailty, or limited life expectancy. However, there is limited evidence on the health benefits and safety of deprescribing for many medications. To make informed and patient-centered (patient priority directed; https://patientprioritiescare.org/) treatment decisions [34–36], healthcare providers need to consider the patients’ condition, functional trajectories, life expectancy, treatment risks and benefits, personal goals and preferences, and previous (de)prescribing experiences [35, 37–40].

The frequency of medication review to assess the safety and effectiveness of older adults' medication regimens is not uniformly recommended. Regular comprehensive evaluations, considering changes in overall health and prognosis, are advisable, particularly for frail individuals [21]. According to National Institute for Health and Care Excellence (NICE) guidance, medication review should ideally occur every 6 months for frail or cognitively impaired older adults [41]. Detailed medication review and deprescribing should also be considered when new symptoms or conditions arise or when ADRs or advanced/end-stage diseases, severe dementia, extreme frailty, or long-term care facility admissions occur [42]. In medication review, careful attention should be given to patient characteristics, drug–disease interactions, and the efficacy and safety of each treatment before initiating, continuing, or discontinuing medications. Additionally, special attention is necessary for high-risk medication combinations, unnecessary or ineffective medications, and preventive drugs for individuals with frailty or limited life expectancy [43].

Over the last decades, many checklists, online resources (e.g., evidence-based deprescribing guidelines at www.deprescribing.org), and guidelines have been developed to support health care providers in the process of (de)prescribing in older people [6, 44–46]. A recent review [47] identified over 70 drug lists, including the Beers Criteria [48], Fit-For-The-Aged (FORTA) [49], the Screening Tool of Older Person’s Prescriptions and Screening Tool to Alert to Right Treatment (STOPP/START) [50], and Turkish Inappropriate Medication Use in the Elderly (TIME) [42]. These lists can be classified as either Drug-Oriented Listing Approaches (DOLAs), or Patient-In-Focus Listing Approaches (PILAs) for which knowledge of patient characteristics is required [47]. In randomized-controlled trials (RCTs) [51–53], PILAs (e.g., FORTA and STOPP/START) were more beneficial to patients than DOLAs [47], especially if they not only identify overtreatment by PIMs, but also positively label drugs or actions on drugs (e.g., dose adjustment) that may have been omitted [potentially omitted medications (POMs)]. The demonstrated clinical benefits in RCTs suggest that deprescribing by itself is not always sufficient to improve an older patient’s clinical status.

### Organizing healthcare systems with focus on deprescribing

Given their expertise and knowledge of older adults with multimorbidity, and age-related alterations in pharmacodynamics and kinetics, geriatricians are in an ideal position to perform medication reviews and optimizing pharmacotherapy (including deprescribing) for this patient population. Thus, geriatricians are urged to take the lead in this area. However, given the large number of older adults involved, geriatrician-led medication reviews cannot be the standard for all and collaboration is warranted. In principle, general practitioners (GPs) should regularly review medications and this should include evaluation of deprescribing opportunities in their older patients. They already are the main prescribers of older adults’ medications [54], have detailed knowledge of their patient’s past and current diagnoses and treatments, are the connection between healthcare providers, and often have a longstanding professional relationship with their patients. The associated level of trust among patients regarding health care professionals’ decisions to change or discontinue (long-term) treatments is considered essential for successful deprescribing attempts [55]. In complex situations where GPs lack confidence or expertise to perform a medication review, and depending on the organization of national healthcare systems, geriatricians, clinical pharmacologists, and/or pharmacists can provide support, or take responsibility for the medication review and represcribing. For effective management of polypharmacy and deprescribing medications, it is recommended to have interprofessional communication on a case-by-case patient level to define the roles and responsibilities between hospital specialists and primary care physicians. Integrating the skills and professional expertise of pharmacists and other healthcare professionals can help to address the medical complexity of older patients [6, 35]. Collaboration among healthcare professionals also allows for shared responsibility and workload associated with deprescribing [10]. Many studies have reported highly effective deprescribing interventions involving pharmacists [7, 56]. Pharmacists are well equipped for tasks such as medication reconciliation, review, patient education, and advising on safer alternative medications. They can also assist in detecting and optimizing adherence [57]. However, deprescribing interventions carried out by pharmacists or physicians may require significant effort, time, and costs, making it challenging to implement in real-world clinical care.

In some countries, nurses can acquire an advanced role as nurse prescribers (also called nurse practitioners), enabling them to play an active role in deprescribing as well, for example in the monitoring phase, by educating and coaching patients and their caregivers and communicating with other healthcare providers [35, 58]. For countries where nurses cannot be actively involved in pharmaceutical care, specialized polypharmacy clinics may be considered [59].

### Deprescribing settings and communication between prescribers

Older patients with polypharmacy and multimorbidity often receive care from multiple healthcare professionals in different specialties and settings. Accurate medication reconciliation is crucial for optimal pharmacotherapy [60]. Effective communication of medication changes and management plans among healthcare professionals is essential for safe and effective polypharmacy management in older adults, particularly during care transitions [61, 62]. However, there is currently a lack of uniform pharmacotherapeutic documentation standards in most European Union (EU) countries. Canadian researchers have recently developed templates for deprescribing recommendations between pharmacists and clinicians, with promising results from pilot trials [63].

To facilitate decision-making for all care providers, we propose a minimal set of data to be recorded in patients' medical records, including the indication and planned duration of treatment, rationale for medication initiation or changes, treatment goals, and patient adherence. Standardized documents should be used to communicate medication changes and management plans, incorporating indications/rationales, treatment goals, planned duration, goals of care, patient preferences, tapering regimens, and monitoring plans for deprescribing attempts.

Comprehensive geriatric assessments by geriatricians should routinely include a review of medication appropriateness and patient willingness to deprescribe. However, deprescribing practices may vary depending on settings and available resources. Geriatric day hospitals, outpatient clinics, rehabilitation centers, and long-term care facilities are suitable for proactive medication-related problem identification based on comprehensive assessments [25, 35]. Deprescribing is also recommended during unplanned hospital admissions, particularly if the admission is related to medications. However, the short duration of hospital stays may limit proper evaluation of therapy modifications and patient recovery. Therefore, geriatricians should provide appropriate instructions to patients and include detailed deprescribing information in discharge letters to ensure awareness and appropriate monitoring by GPs and other treating physicians. Experts propose systematic screening of older patients' willingness to deprescribe, similar to falls or depression screening, possibly using tools such as the Patients' Attitudes Towards Deprescribing (PATD) questionnaire [64], which can inform clinicians about patient preferences and facilitate patient-centered decision-making (Table 1).

**Table 1:** Clinical practice recommendations

*Performing medication reviews:*	
-Regular comprehensive evaluations of older adults' medication regimens should be conducted, considering changes in overall health and prognosis, particularly for frail individuals. For frail or cognitively impaired older adults, medication review should ideally occur every 6 months	
-Medication review in older adults should be performed by general practitioners in collaboration with pharmacists and geriatricians. In selected patients, and depending on the organization of national healthcare systems, geriatricians, clinical pharmacologists and/or pharmacists can provide support, or take responsibility for the medication review and represcribing as required	
-Structured and patient-centered medication review—including appropriate deprescribing—should be a standard component of a comprehensive geriatric assessment	
*Consider individual patient characteristics: *	
Healthcare providers need to consider the patients' condition, functional trajectories, life expectancy, treatment risks and benefits, personal goals and preferences, and previous (de)prescribing experiences when making treatment decisions	
*Utilize available resources: *	
Use structured and validated tools (e.g., evidence-based deprescribing guidelines, clinical decision support systems and listing approaches such as STOPP/START) to assist in in identifying potentially inappropriate medications (PIMs) and potentially omitted medications (POMs)	
*Collaborate among healthcare professionals: *	
Interprofessional communication and collaboration among healthcare professionals, including general practitioners, geriatricians, clinical pharmacologists, pharmacists, and nurses, can help address the medical complexity of older patients. Shared responsibility and workload associated with deprescribing should be encouraged	
*Standardize documentation and communication: *	
Standardized documents should be used to communicate medication changes and management plans, incorporating relevant information such as indications/rationales, treatment goals, planned duration, goals of care, patient preferences, tapering regimens, and monitoring plans for deprescribing attempts	
*Incorporate patient preferences: *	
Clinicians should assess patients' attitudes towards deprescribing. Patient-centered decision-making should include considering patient preferences and goals. Medication review in older adults should be performed by general practitioners in collaboration with pharmacists on a regular basis	

## Organizational factors, implementation, and policy

To facilitate appropriate drug prescribing, country-specific and the most comprehensive up-to-date explicit national criteria of PIMs are required [5]. At present, in some EU countries (e.g., The Netherlands, Denmark, Germany, and Italy), national implicit guidelines for managing polypharmacy and deprescribing have been published. These evidence-based guidelines assist healthcare providers in clinical decision-making and rational deprescribing practices. Efforts should be made to develop such guidelines for countries lacking these. This may result in better patient and policy maker engagement, bringing deprescribing into the routine care dialogue [65]. Also, we advocate the need for more information/guidance on deprescribing in (inter)national “disease-specific” guidelines that so far emphasize medication prescription over deprescribing. Finally, to improve (de)prescribing across Europe, regulatory aspects related to PIM approvals, marketing, and availability should be harmonized and better regulated. At present, there are considerable cross-country differences in approval rates, marketing, recommendations, and preferences for the use of PIMs [5]. Special focus should be on a limited set of high-risk medications [48]. In addition, it is desirable that regulatory agencies [for example the European Medicines Agency (EMA)] prioritize deprescribing, and include recommendations for withdrawing medications, instead of merely recommendations when to start medications.

Current healthcare systems are highly fragmented, consisting of numerous disciplines and organizations. There is limited time and resources available for deprescribing [66], and prescribing quality indicators are lacking [67]. These factors, combined with public and healthcare provider-related factors, contribute to the complexity of reducing inappropriate polypharmacy.

Some countries have concluded that a coordinated national action plan is required to make the needed transformation (lown-eliminating-medication-overload-web.pdf (lowninstitute.org). For example, in Australia, over 100 stakeholders collaborated to develop a national strategic action plan for reducing inappropriate polypharmacy in older people and the following items were included: (1a) exploring opportunities to update documents that guide medication use to explicitly include issues of multimorbidity, polypharmacy, and deprescribing; (1b) investigate the inclusion of a mandatory section to the approved product information consumer medication information of all medications titled ‘Cessation’ or ‘Deprescribing’; (2) integrate health care to provide multi-disciplinary patient-centered pharmaceutical care; (3) collect and use health data to monitor and address polypharmacy at the community, health care professional and consumer level; (4) provide incentives to health care professionals for optimizing quality use of medicines by older adults; (5) provide health care professionals with education and tools to optimize quality use of medicines by older patients; (6) raise consumer awareness of polypharmacy and deprescribing and provide tools to help consumers discuss the issues with their prescribers, and (7) develop a national strategic plan for research on polypharmacy and deprescribing [68].

Many older adults are unaware that certain medications may be harmful [69, 70], and that deprescribing inappropriate medications or switching to safer alternatives may be possible. Improving older adults’ health literacy has been shown to be a valuable intervention to reduce the harms associated with inappropriate polypharmacy [20]. Patient and public engagement is essential for managing polypharmacy and deprescribing PIMs. Therefore, public awareness should be increased [69] making deprescribing more common [55, 56, 65, 71]. There have been various large-scale programs aimed to facilitate the deprescribing movement by targeting the public [72]. For example, in the D-PRESCRIBE study [28], brochures about high-risk medications were e-mailed to patients. In addition, the patients’ primary care physician received a document, containing evidence-based rationale and options for stopping medications when appropriate. This intervention resulted in a reduction in sedative use of 43% over 6 months, with a number needed to treat (NNT) of only three. Another example is a regional public awareness campaign in Southern Australia about the benefits and harms of benzodiazepines, and the availability of non-pharmacological alternatives. This initiative produced a 19% reduction over 2 years in benzodiazepine dispensing [73]. Furthermore, receipt of a mailed educational booklet outlining the benefits and harms of chronic benzodiazepine use for insomnia yielded a benzodiazepine discontinuation rate of 27% compared with 5% in the control group 6 months after the intervention [74]. In Europe, enhancing patient empowerment towards safer prescribing is one of the main aims of the recently launched SafePolyMed program (Table 2).

**Table 2:** Organizational, implementation, and policy recommendations

*Active role for geriatricians:*	
Depending on the organization of national healthcare systems, geriatricians or experts in geriatric/gerontological pharmacology should be actively involved in developing (inter)national or local plans to improve safe (de)prescribing in older adults with multimorbidity, for example by collaborating with international healthcare professionals and researchers in deprescribing networks.	
*Develop country-specific explicit and implicit national criteria for potentially inappropriate medications (PIMs) and deprescribing: *	
-For each country, comprehensive and up-to-date guidelines that explicitly define PIMs should be established.	
Efforts should be made to develop evidence-based guidelines for managing polypharmacy and deprescribing in countries that currently lack them.	
*Incorporate deprescribing in disease-specific guidelines:*	
Existing disease-specific guidelines should include information and guidance on deprescribing to ensure that deprescribing is given equal importance and consideration in the management of specific health conditions as prescribing.	
*Harmonize regulatory aspects related to PIMs: *	
Regulatory agencies should work towards harmonizing the approval, marketing, and availability of PIMs across different countries. This includes prioritizing the withdrawal of medications and providing recommendations for discontinuation, in addition to recommendations for starting medications.	
*Establish a coordinated national action plan:*	
-Countries should develop their own national action plans for multimorbidity and polypharmacy management, providing at minimum.	
-A strategy to engage patients and caregivers in deprescribing, and increase their awareness on potential adverse drug events (e.g., falls), PIMs, and altered risk/benefit ratios. An important goal to communicate is to emphasize the potential benefits of deprescribing.	
-Endorsement of structured approaches for medication optimization based on listing approaches.	
-A plan for educating healthcare professionals to optimize prescribing and deprescribing.	
-A plan to provide incentive or remuneration for deprescribing, and to provide sufficient organizational and financial support for multi-disciplinary care and non-pharmacological alternatives.	
*Improve health literacy, public awareness, and public engagement:*	
-Efforts should be made to improve older adults' health literacy to increase their awareness of potentially harmful medications and the possibility of deprescribing.	
-Engaging patients and the public in the process of managing polypharmacy and deprescribing is essential.	

## Education and training

### Undergraduate training

To meet the challenges and demands of an ageing population, we need to adapt undergraduate education and ensure that all newly qualified doctors are well equipped to care for older patients with complex health and social care needs [75], including managing polypharmacy and deprescribing [76, 77]. At present, teaching polypharmacy and deprescribing are still an evolving topic in undergraduate curricula. Based on experts from the European Union of Medical Specialists-Geriatric Medicine Section (EUMS-GMS) board, the Education SIG of the EuGMS [76], and the International Union of Basic and Clinical Pharmacology (IUPHAR), managing polypharmacy, deprescribing and minimizing low-value care (continuation of potentially futile medications associated with ADRs) should receive priority [78].

For effective and personalized pharmacotherapy (including deprescribing), a patient-centered, multi-disciplinary approach, adequate knowledge, attitudes, and communication skills are required, for which students should be adequately trained. First, the learning outcomes of medical students should include recognition of frailty, which is crucial in medication-related decision-making in older individuals [78]. Also, knowledge of deprescribing tools (e.g., Screening Tool of Older Persons Prescriptions in Frail Adults with Limited Life Expectancy (STOPPFrail) [79]), age- and frailty-related changing risk management and treatment goals should be taught. Technical knowledge should be provided to facilitate rational and safe pharmacotherapy, including NNT, number needed to harm (NNH), time-to-benefit (TTB), ADRs, optimal tapering strategies, and non-pharmacological treatment options. Competencies for shared decision-making, communication, and managing health systems should be included in the training. Ideally, future doctors should be trained for optimal use of terminology: instead of telling patients they “need” medications for a certain benefit, or telling the medication is “for the rest of their lives”, they should discuss the risks of a medication up-front [55], and consider the use of safer (non-pharmacologic) alternatives. To prevent prescribing cascades (a new medicine is prescribed to 'treat' an adverse reaction to another drug) [80, 81], students should be taught to have a high level of suspicion for new symptoms to be an adverse effect of other medications that were recently initiated in older people with polypharmacy [82].

Future doctors should be taught communication skills to be able to openly discuss current medication adherence, satisfaction, and preferences with their patients, and discuss their willingness to deprescribe [83]. In deprescribing discussions, optimizing therapy and minimizing risk of adverse effects should be emphasized [37]. Also, deprescribing attempts should be framed as a trial, reassuring patients that medication can be re-initiated whenever necessary [84]. The importance of exploring the patients’ willingness to deprescribe should be emphasized, as opposed to assuming that patients may not be willing to deprescribe a longstanding medication [85]. Indeed, literature consistently shows that clinicians (including geriatricians [86]) perceive patients to be unwilling to deprescribe their medications, despite evidence indicating the opposite: the majority of geriatric patients and nursing home residents want to take fewer medicines, and are hypothetically willing to stop a medicine on their clinician’s recommendation [20, 35, 87]. Also, students should be trained to communicate uncertainty with patients: in older adults with multimorbidity, benefits and harms of both prescribing and deprescribing medications are often uncertain, stressing the need for an individualized, holistic approach that is driven by the patients’ goals and preferences for care, and requires clinical judgement [42]. Finally, students should be encouraged to be proactive in reviewing polypharmacy and optimizing medication, especially in geriatric and palliative care patients.

Ideally, education on optimization of pharmacotherapy and deprescribing should include interprofessional educational approach where students from two or more health or social care professions learn interactively together with the aim of providing high-quality, patient-centered care [76]. It is beneficial for students, because it not only expands knowledge, but they also learn about roles and responsibilities, and effective communication as a team [76]. Given their specific knowledge, expertise, and skills, we advocate that experts on geriatric prescribing and/or pharmacotherapy are actively involved in the development of undergraduate educational programs on polypharmacy and deprescribing, not only for medical students, but also for student pharmacists, nurses, and other healthcare professionals.

Geriatricians and geriatricians-in-training often report that they received insufficient education and training in polypharmacy management and deprescribing in medical school [86]. In another study, hospital clinicians reported limited self-efficacy in deprescribing [88]. Recently, European societies have defined a common core curriculum with a list of minimum training requirements for obtaining the specialty title of geriatric medicine [89]. According to that listing [90], geriatric trainees should be able to: “explain the indications and contraindications, mechanism of action, effectiveness, potential adverse effects, potential drug interactions, and alternatives for medications commonly used in older patients. They should also be able to recognize symptoms that could be explained by ADRs and risk factors for increased risk of ADRs. Knowledge of the basic principles of drug-drug interactions, drug-food interactions, and effects of disease states on drug pharmacokinetics is important. Trainees should acquire knowledge on polypharmacy, PIMs, and under- or overuse of the most common drugs in older patients”. In addition, it is stated that “Geriatricians entering into unsupervised practice, in and across all care settings, are able to: provide comprehensive medication review to maximize benefit and minimize number of medications and adverse events”.

Specialized nurses in geriatrics and/or long-term care facilities should be trained for their role in monitoring adverse effects, assessing adherence, and providing non-pharmacological patient education [36] at least in countries where these healthcare professionals are allowed to prescribe medications. Their education should include knowledge on non-pharmacological interventions, for example regarding challenging behavior/behavioral and psychiatric symptoms of dementia [90, 91].

### Postgraduate training

International experts in the field of deprescribing recommend education on optimal prescribing and deprescribing continuing from the undergraduate level through to continuing professional development [55]. We recommend that educational material is developed for European geriatricians to increase their knowledge on medication review, polypharmacy, and deprescribing. To this end, a Massive Open Online Course (MOOC) could be developed. MOOCs are free-of-charge (“open”) online training courses that can be used to disseminate knowledge and skills to a large amount of individuals (“massive”) [92], as has been successfully done by the “Screening for Chronic Kidney Disease among Older People across Europe” consortium [92].

Given their specific knowledge, expertise, and skills, we advocate that experts on geriatric prescribing and/or pharmacotherapy are actively involved in the development of postgraduate educational programs on polypharmacy and deprescribing, not only for medical doctors, but also for pharmacists and specialized nurses working in the geriatric field (Table 3).

**Table 3:** Education and training recommendations

*Adapt undergraduate education:*	
-Incorporate basic training on managing polypharmacy and deprescribing in undergraduate medical education to ensure that all newly qualified doctors are well equipped to care for older patients with complex health and social care needs.	
-The following topics should receive priority in education:	
Specific knowledge on deprescribing steps and tools, and of age- and frailty-related drug risk/benefit ratios.	
Managing polypharmacy, deprescribing, and minimizing low-value care, particularly in geriatric and palliative care patients.	
Frailty recognition, and knowledge of frailty-related changing risk management and treatment goals as a crucial aspect of medication-related decision-making in older individuals.	
Technical knowledge to facilitate rational and safe pharmacotherapy, including concepts such as Number Needed to Treat (NNT), Number Needed to Harm (NNH), time-to-benefit, adverse drug reactions, optimal tapering strategies, prescribing cascades and non-pharmacological treatment options.	
Communication skills, including decision-making, effective communication with patients regarding medication risks, and exploring patients' willingness to deprescribe.	
*Foster interprofessional education: *	
An interprofessional educational approach should be adopted, where students from various healthcare professions (e.g., medical students and pharmacy students) learn together, promoting teamwork and effective communication.	
*Offer advanced education and training in geriatric specialty programs: *	
Geriatricians-in-training should receive advanced education and training in managing polypharmacy and deprescribing.	
*Develop postgraduate continuing education opportunities: *	
Extend education on optimal prescribing and deprescribing from the undergraduate level through to continuing professional development. Offer educational materials and courses, such as a Massive Open Online Course (MOOC), to increase the knowledge of healthcare professionals in geriatric prescribing.	
*Involve experts in curriculum development: *	
To ensure the inclusion of specialized knowledge and skills in the curriculums, engaging experts in geriatric prescribing and/or pharmacotherapy in the development of undergraduate and postgraduate educational programs on polypharmacy and deprescribing should be encouraged.	

## Research

### Clinical trials

Frail older individuals are often underrepresented in clinical trials, and functional outcomes are frequently overlooked, particularly in trials involving medications commonly prescribed to this population [93]. This underrepresentation presents a significant barrier to the application of modern epidemiological techniques and the expansion of our knowledge base. To enable healthcare professionals to make informed prescribing decisions for older individuals with multimorbidity and polypharmacy, it is crucial to enhance our evidence base concerning the potential harm and benefits of (de)prescribing medicines in older adults with multimorbidity. This includes investigating metrics such as the NNH, NNT, efficacy of non-pharmacologic alternatives to PIMs [94], and adverse drug withdrawal events (ADWEs). Consequently, prioritizing funding for studies focusing on medications with an overall positive benefit/risk ratio in older adults is essential. Key outcomes to focus on should include patient-centered goal attainment, TTB approaches, and de-escalation trials, particularly within the fields of geriatric oncology and hematology [95]. Ideally, these trials should have longer durations and incorporate baseline functional parameters. Additionally, real-world evidence studies allow for the evaluation of the representativeness of RCT data in real-life patient populations. Furthermore, such studies provide opportunities to generate comparative safety and efficacy data.

### Existing tools

A multitude of tools have been developed, and various online resources are available to assist clinicians in achieving appropriate (de)prescribing practices. However, it is recommended to validate, refine, and regularly update these existing tools before considering the development of new ones. Additionally, it is essential to adapt these tools to local settings, taking into account the differences in registered medications and prescribing practices among regions and countries [96].

### Deprescribing trials

A growing number of deprescribing trials are being conducted, and recent systematic reviews show an effect in terms of decreased prescribing of PIMs [29, 97]. However, strong evidence for consistent and sustainable changes in clinical outcomes is still lacking. This can be explained by methodological limitations. Scott et al. listed the following items potentially explaining study limitation factors for RCTs, namely: small sample size, short follow-up time, infrequent use of quality-of-life measures, insufficient targeting of patients at highest risk of medication-related harm, suboptimal intensity or duration of deprescribing interventions, and limited use of potentially useful clinical decision support system (CDSS) to assist deprescribing [33]. In the United States, the Food and Drug Administration (FDA) has launched a new road map 2030 for drug evaluation in older adults. The road map contains support for deprescribing studies [98]. In contrast, the EMA does not have a comparable agenda and has not reopened the Geriatric Expert Group.

Implementing deprescribing in clinical trials has proven to be challenging. For instance, recent large European trials on medication optimization, such as SENATOR [99] and OPERAM [100], reported disappointing acceptance rates of deprescribing advice. When developing new interventions, it is important to consider and address the barriers and facilitators to deprescribing at various levels, including individuals and the public, healthcare professionals, and the healthcare system [66].

In addition to suboptimal implementation, the low intensity of interventions may partially account for the negative outcomes observed in the majority of deprescribing trials [33]. Relying solely on a single medication review conducted by a pharmacist or physician might not be sufficient. Instead, healthcare professionals and patients require adequate time, interactions, information, and motivation to develop, agree upon, initiate, and monitor deprescribing decisions [33]. For instance, in the OPERAM trial [100], the single review was proposed as one of the explaining factors for negative findings as contacts with other doctors might have led to changes in medication regimens. Naturally, in a busy clinical practice, it is crucial to find the right balance between the feasibility and intensity of the intervention.

In addition, several recommendations were not implemented during the hospital admission as some hospitalized patients first wanted to consult their GP about the medication change recommendations [100]. Moreover, several STOPP/START criteria might not be relevant during acute hospital admission [99]. These examples highlight the importance of the timing of the medication review in future trials and the adjustment of the used tool to the setting [101]. In addition, follow-up duration should be longer to ascertain potential long-term deprescribing effects.

It should be stressed that deprescribing interventions are complex interventions, comprising a number of interactions between components, different stakeholders/users, variable outcomes, and a number of uncertainties. The updated Medical Research Council (MRC) framework can guide the development and evaluation of such interventions [102]. This framework can help design interventions that fit within clinician’s workflow and engage all stakeholders, including patients and professionals in each step (develop or identify intervention, feasibility assessment, evaluation, and implementation). Furthermore, to understand the feasibility and acceptability of different deprescribing interventions, common implementation outcomes should be developed for deprescribing trials [103].

The use of electronic medical records (EMRs) provides an opportunity to develop and utilize CDSSs that could incorporate deprescribing tools. Although the potential to reduce PIM prescribing through EMR-enabled CDSSs has been demonstrated in hospitalized older adults [104], evidence that this translates to improved clinical outcomes is limited. Literature suggests that improvement in clinical outcomes (e.g., reduction in adverse drug reactions) can be achieved if geriatricians are involved in the intervention [105, 106]. In line with this, it should be emphasized that CDSS-based approaches are always part of multicomponent complex interventions. They require careful development of the CDSS in collaboration with the end-users, and adequate integration in the workflow of the clinicians is essential. Also, adequate educational and implementation efforts supported by specific end-user expertise are warranted. Ultimately, CDSSs should be seamlessly integrated into EMRs at the point of care, user-friendly, and responsive to patient contexts to avoid alert fatigue [107].

To date, inconsistent and heterogeneous outcome definitions of deprescribing have been used [103]. To enable comparison and synthesis of trial results, a Core Outcome Set (COS) should be used as these provide a base for robust evidence regarding deprescribing research. COS is a consensus minimum set of standardized outcomes to be used in all trials of a specific field. Currently, COS exists for medication reviews in older people with polypharmacy, for addressing polypharmacy in older people in primary care, and for deprescribing in hospital for older people [19, 108, 109]. Furthermore, a future framework for deprescribing trials should focus on patient-centered outcomes [110].

Non-inferiority designs have been proposed as an alternative to classical superiority analysis. It is important to consider the clinical, financial, and economic aspects that influence deprescribing decisions. Considering the decrease in drug burden and costs, a lack of change in clinical status (e.g., functioning or symptoms) following deprescribing could be considered a positive outcome. Non-inferiority designs can help evaluate whether deprescribing leads to "no change" [111, 112]. Another study design that may be considered for addressing polypharmacy and deprescribing is the n-of-1 trial. N-of-1 trials involve crossover experiments within individual patients, allowing for a comparison of the effects of continuing with the current treatment versus no treatment or placebo [113, 114]. N-of-1 trials are appealing as they generate patient-specific evidence that can inform deprescribing decisions and promote therapeutic precision [114]. Considering the challenges associated with conducting RCTs on deprescribing, the use of observational research using large administrative datasets, such as electronic health records or claims data, could be considered as an additional data source to support the evaluation of specific deprescribing research questions within RCTs [115]. In recent decades, significant advancements have been made in pharmaco-epidemiology and comparative effectiveness research, including the emulation of target trials, active comparator new user designs, and the use of prior event rate ratios or propensity scores, which can be applied to deprescribing research [115]. To employ comparative effectiveness research in the field of deprescribing, rich data for large numbers of individuals are necessary [115]. However, the possibility for unmeasured confounding still exists.

There is a significant knowledge gap regarding the heterogeneous treatment effects of deprescribing, as highlighted by Scott et al. [33]. Individual responses to PIMs and deprescribing can vary due to inter-individual differences, such as age, frailty, and comorbidities. Future research efforts should aim to identify which individuals benefit most from deprescribing and who are most likely to re-initiate medication after deprescribing [33].

To optimize interventions and target individuals at the highest risk of adverse drug events, it is important to consider sex/gender-related pharmacokinetic (PK) and pharmacodynamic (PD) differences. Despite women being the largest consumers of medications and facing an increased risk of ADRs compared to men, existing research has largely overlooked this consideration [3]. Therefore, there is a pressing need for more research focusing on the influence of sex and gender on inappropriate prescribing and deprescribing [3] (Table 4).

**Table 4:** Future research

More research is needed on how and when to deprescribe, and on possible effects of deprescribing.	
*Clinical trials: *	
-Prioritize funding for studies focusing on medications with an overall positive benefit/risk ratio in older adults.	
-Incorporate patient-centered goal attainment and time-to-benefit approaches as key outcomes in trials involving older adults with multimorbidity.	
-Conduct longer duration trials that incorporate baseline functional parameters to assess the efficacy of interventions.	
-Utilize real-world evidence studies to evaluate the representativeness of randomized-controlled trial (RCT) data in real-life patient populations and generate comparative safety and efficacy data.	
*Existing tools: *	
-Refrain from developing new tools, but validate, refine, and regularly update existing tools for appropriate (de)prescribing practices.	
-Adapt these tools to local settings, considering the differences in registered medications and prescribing practices among regions and countries.	
*Deprescribing interventions:*	
-Address methodological limitations (e.g., small sample size, infrequent use of quality-of-life measures), and consider non-inferiority designs and n-of-1 trials as alternative study designs.	
-The updated framework of Medical Research Council (MRC) can guide the development and evaluation of deprescribing interventions.	
-Consider high-risk patients for medication-related harm and optimize intensity and duration of deprescribing interventions.	
-Develop and utilize clinical decision support systems (CDSSs) integrated into electronic medical records (EMRs) to assist deprescribing, while involving end-users in the development process.	
-Use Core Outcome Sets (COS) to standardize outcome definitions in deprescribing research and focus on patient-centered outcomes.	
-Explore the use of observational research using routine datasets to supplement randomized-controlled trials (RCTs).	
-Investigate heterogeneous treatment effects of deprescribing and identify individuals who benefit most from deprescribing.	
-Consider sex (biological) and gender (socio-cultural) differences when designing deprescribing interventions.	

## Conclusion

In summary, the EuGMS SIG on Pharmacology advocates a multifaceted and wide-scale change in education, guidelines, research, advocacy, and policy to improve the management of polypharmacy in older people, and to make deprescribing part of routine care for the ageing generations to come. In our opinion, it is important that geriatricians and experts in geriatric/gerontological pharmacology are in the lead and actively take part in this change.

## Abbreviations

ADR: Adverse drug event
ADR: Adverse drug reaction
ADWE: Adverse drug withdrawal event
CDSS: Clinical decision support system
COS: Core outcome set
DOLA: Drug-oriented listing approach
EMA: European Medicines Agency
EU: European Union
EMR: Electronic medical record
EuGMS: European Geriatric Medicine Society
EUMS-GMS: European Union of Medical Specialists-Geriatric Medicine Section
FDA: Food and Drug Administration
FORTA: Fit-fOR-The-Aged
GP: General practitioner
IUPHAR: International Union of Basic and Clinical Pharmacology
MOOC: Massive Open Online Course
MRC: Medical Research Council
NICE: National Institute for Health and Care Excellence
NNH: Number needed to harm
NNT: Number needed to treat
PATD: Patients' Attitudes Towards Deprescribing
PD: Pharmacodynamic
PILA: Patient-in-focus listing approach
PIM: Potentially inappropriate medication
PK: Pharmacokinetic
POM: Potentially omitted medication
RCT: Randomized-controlled trials
SAE: Serious drug event
SIG: Special Interest Group
STOPPFrail: Screening Tool of Older Persons Prescriptions in Frail Adults with Limited Life Expectancy
STOPP/START: Screening Tool of Older Person’s Prescriptions and Screening Tool to Alert to Right Treatment
TIME: Turkish Inappropriate Medication Use in the Elderly
TTB: Time-to-benefit
WHO: World Health Organization
